# Strength exercises during physical education classes in secondary schools improve body composition: a cluster randomized controlled trial

**DOI:** 10.1186/s12966-018-0727-8

**Published:** 2018-09-25

**Authors:** G. A. Ten Hoor, G. M. Rutten, G. J. P. Van Breukelen, G. Kok, R. A. C. Ruiter, K Meijer, S. P. J. Kremers, F. J. M. Feron, R. Crutzen, A. M. J. W. Schols, G. Plasqui

**Affiliations:** 1 Department of Human Biology and Movement Sciences, NUTRIM School of Nutrition and Translational Research in Metabolism, Maastricht University Medical Centre+, P.O. Box 616, 6200 MD Maastricht, The Netherlands; 2 Department of Work and Social Psychology, Maastricht University, P.O. Box 616, 6200 MD Maastricht, The Netherlands; 3 Department of Health Promotion, NUTRIM School of Nutrition and Translational Research in Metabolism, Maastricht University Medical Centre+, P.O. Box 616, 6200 MD Maastricht, The Netherlands; 4 Department of Methodology and Statistics, CAPHRI, Care and Public Health Research Institute, Maastricht University, P.O. Box 616, 6200 MD Maastricht, The Netherlands; 5 Department of Social Medicine, CAPHRI, Care and Public Health Research Institute, Maastricht University, P.O. Box 616, 6200 MD Maastricht, The Netherlands; 6 Department of Health Promotion, CAPHRI, Care and Public Health Research Institute, Maastricht University, P.O. Box 616, 6200 MD Maastricht, The Netherlands; 7 Department of Respiratory Medicine, NUTRIM School of Nutrition and Translational Research in Metabolism, Maastricht University Medical Centre+, P.O. Box 616, 6200 MD Maastricht, The Netherlands

**Keywords:** Strength exercises, Overweight and obesity, School-based, Cluster randomised controlled trial, Body composition, Motivation, Physical activity

## Abstract

**Background:**

Metabolic health in people with obesity is determined by body composition. In this study, we examined the influence of a combined strength exercise and motivational programme –embedded in the school curriculum– on adolescents body composition and daily physical activity.

**Methods:**

A total of 695 adolescents (11-15y) from nine Dutch secondary schools participated in a one year cluster randomised controlled trial (RCT). In the intervention schools, physical education teachers were instructed to spend 15–30 min of all physical education lessons (2× per week) on strength exercises. Monthly motivational lessons were given to stimulate students to be more physically active. Control schools followed their usual curriculum. The primary outcome measure was body composition assessed by the deuterium dilution technique. Daily physical activity and sedentary behaviour measured by accelerometry served as a secondary outcome.

**Results:**

After 1 year, a 1.6% fat mass difference was found in favour of the intervention group (*p* = .007). This reflected a 0.9 kg difference in fat free mass (intervention>control*; p* = .041) and 0.7 kg difference in fat mass (intervention<control; *p* = .054). Daily physical activity decreased from baseline to posttest in both groups, but less so in the intervention group (*p* = .049). After 1 year, a difference of 0.4% was found for moderate to vigorous physical activities in favour of the intervention group (*p* = .046). No differences in sedentary behaviour, or light physical activity were found between groups.

**Conclusion:**

In 11–15 year olds, the combination of strength exercises plus motivational lessons contributed to an improvement in body composition and a smaller decrease in physical activity level.

**Trial registration ID:**

(NTR5676 – retrospectively registered 8 February 2016; enrolment of first participant: 2 March 2015).

**Electronic supplementary material:**

The online version of this article (10.1186/s12966-018-0727-8) contains supplementary material, which is available to authorized users.

## Background

Obesity, defined as an excessive body fat percentage, is a global health problem [1, 2] and an important risk factor for chronic metabolic and cardiovascular diseases [3, 4]. Although prevalences fluctuate per country (and plateaued in high income countries), the mean BMI and prevalence of obesity has increased worldwide [1]. Nowadays, more and more children are obese, and without preventive measures, a child’s unhealthy weight is likely to be sustained in later life [3, 5]. In the Netherlands, 13.6% of 4–17 year olds are overweight, and 2.7% are obese [6].

Besides overeating and genetic susceptibility, an insufficient level of physical activity and a high level of sedentary behaviour are some of the main contributors to childhood overweight and obesity [7], and the target of many obesity reduction programmes [8]. These programmes often focus on 1) limiting calory intake and eating healthier and/or 2) improvements in the daily physical activity pattern. However, while quickly reducing weight by calory restriction may result in short term success, weight is often regained rapidly as a result of several mechanisms (e.g.by dieting, one’s energy expenditure adjusts to a lower energy intake making long term dieting a necessity for the maintenance of the lost weight, hormonal changes, increased neural responsivity, and ‘stressed fat cells’- see also [9]). In the long term, sustainable improvements in daily physical activity are often difficult to achieve for people who are overweight or obese: negative experiences (e.g. lower performance in comparison to others, lack of skills) may result in loss of motivation, causing the individual to further disengage from physical activity. This may contribute to an increase in weight, which in turn may result in even lower levels of physical activity [10, 11].

Attempts to increase physical activity in children or adolescents often focus on aerobic exercises. However, it is increasingly suggested that strength exercises should be incorporated into a child or adolescents’ daily life [10, 11]. These exercises can improve body composition, i.e. an increase fat free mass and/or reduction in fat mass percentage. A higher fat free mass results in an increase in basal metabolic rate and total energy expenditure. In addition, a lower fat mass percentage improves several cardiovascular risk factors [12]. There may also be psychological advantages associated with strength exercises, particularly for overweight youngsters who are generally stronger and will outperform their normal-weight peers during strength tests, increasing compliance and enjoyment [10–13], which can in turn contribute to higher participation in physical activity after school [11].To reduce stigma, and to optimally use social comparison, the focus in the currently presented study was not on weight or adolescents with overweight or obesity, but on health, and on all youngsters.

In this cluster RCT, we investigated the one-year efficacy of incorporating strength exercises into gym classes, in combination with monthly motivational lessons (to engage in physical activities after school) on the body composition and activity level of adolescents.

## Methods

### Study design

**Fig. 1:**
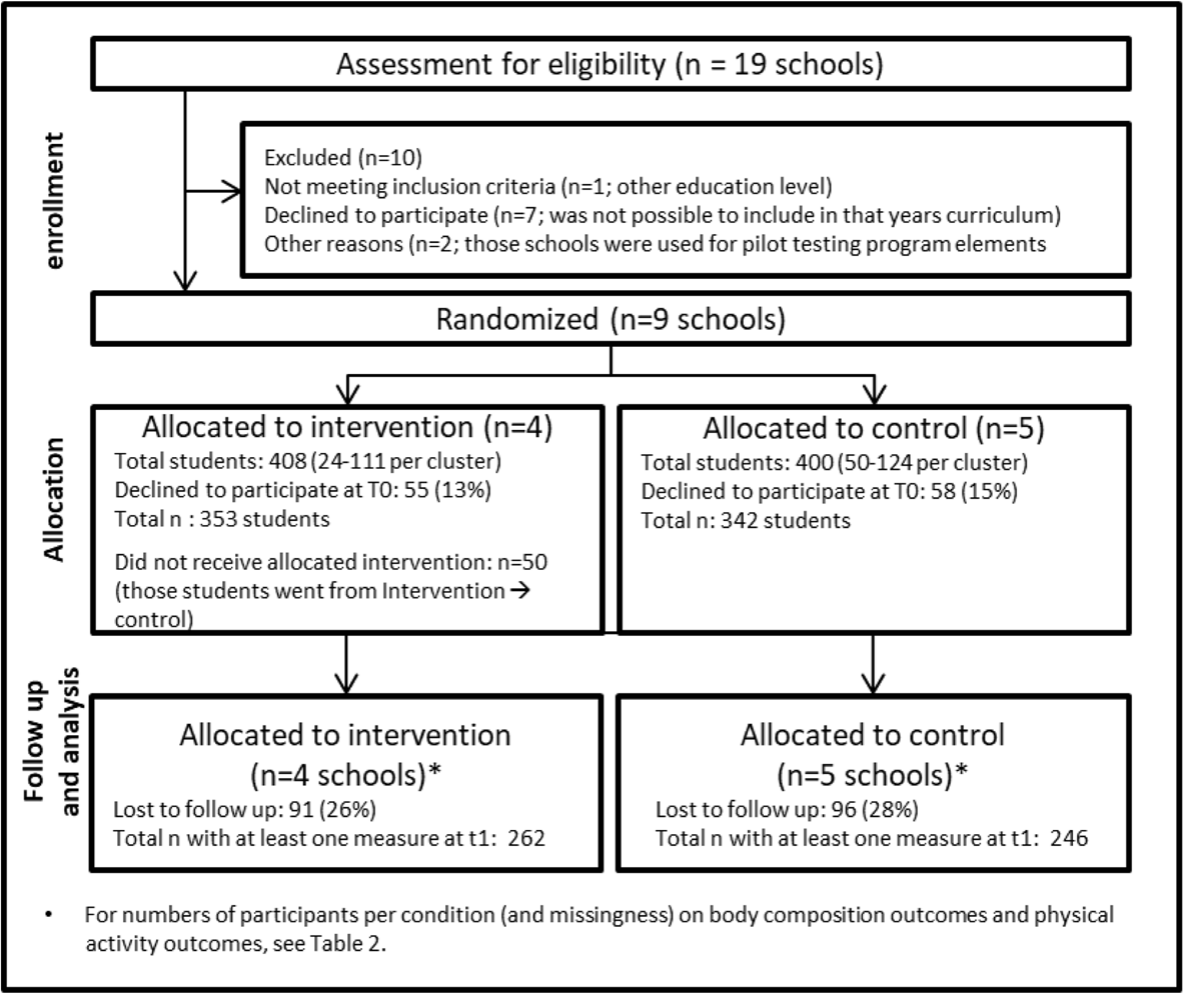
Flowchart of participant selection

Nine Dutch secondary schools (seven schools with Lower Vocational Education, two schools with Senior General Secondary Education) were randomised (stratified on education level; by flip of a coin by the first author under supervision of the fourth author) into an intervention condition (four schools) or a standard curriculum control condition (five schools) (see also Fig. 1). The intervention period was between March 2015 and March 2016. Measurements were taken before (T0) and directly after (T1) intervention. Trial registration ID: NTR5676 (http://www.trialregister.nl/trialreg/admin/rctview.asp?TC=5676).

### Study population

Sample size calculations were performed based on body composition (fat mass percentage) improvements after 12 months for the intervention schools compared to the control schools. With α = 0.05, power = 0.90, and an assumed small to medium effect size (d = .35), 172 participants per group would be needed for a classical RCT. However, in view of the clustering of students within schools and randomised assignment of schools (cluster randomised trial), we aimed for a sample size of 600 to adjust for the design effect arising from clustering [14]. The sample size was further increased to 700 to accommodate 15% dropout (although all available data from all participants would be included into the analysis).

Schools were recruited via school management and 695 adolescents (11–15 years old) participated. Following consent from the schools, parents and their children were informed about the intervention and related outcome measurements, and told they could refuse participation at any time. The study methods and consent procedure were approved by the Ethical Review Committee of the Faculty of Psychology and Neuroscience, Maastricht University, the Netherlands [ERCPN-05-09-2012A1].

### Study interventions

The intervention group received both a strength exercise intervention and a motivational intervention to promote after school physical activity, while the control group continued with their usual curriculum. For an elaborate description of the intervention, see our designpaper by Ten Hoor et al. [15] (open access).

#### Strength intervention

The PE teachers in the intervention group spend at least 30% of the physical education lessons on strength exercises (approximately 15–30 min per lesson – students have 3 h of PE per week),. This proportion was based on the feasibility of integrating strength exercises into the standard curriculum (see also an inspirational workbook in Additional file 1). The teachers were instructed about the program and the specific strength exercises (see Table 1), participated in workshops to improve their motivational speaking skills, and were provided with materials (medicine balls, elastic bands, free weights) to make them able to include strength exercises in their lessons. Furthermore, teachers receive a book with strength exercises and games as inspirational material. This inspirational material was based on literature, ideas from experts in the field, and from the PE teachers themselves (see Additional file 1 for the complete Dutch book). Besides this information package (including: at least 15 min per lesson – 45 min per week; the focus on absolute strength exercises [see Table 1]; and safety guidelines [including good techniques before increasing weight, never maximal strength exercises, no more than 3 × 15 repetitions, enough variation]), teachers were allowed to give their own interpretation of these 15 min.

**Table 1:** The right strength exercises – relative versus absolute

Although the reasoning is simple, not all strength exercises are appropriate. Heavier people are stronger in *absolute* sense. This means that strength exercises where someone’s own body weight plays a role (so called *relative* strength exercises like doing push-ups) can still be experienced as something negative. *Absolute* strength exercises (like strength exercises with free weights) can result in the intrinsic reward that is absent in other physical activities.	
An extra dimension that makes strength exercises more fun, is when being valued based on qualities. A strength exercise where peers say “you win because you are fat” (like tug of war) is less motivating compared to an exercise with free weights or medicine balls where the reaction is “you are good because you are strong!”	

#### Motivational intervention

Once a month, a one-hour lesson was used to increase motivation to be more physically active (see Table 2, our design paper by Ten Hoor et al. [15] (open access) or Additional file 1 for an overview of these lessons, including the Dutch workbook). These motivational lessons were based on motivational interviewing [16] and facilitated by a trained mentor or PE teacher. In the first five months, an extra monthly online motivational lesson was given. In Table 2, an overview of each lesson including its content is given.

**Table 2:** Content per motivational lesson

Lesson	Class/Online	Topic	Motivational Interviewing
1	C	- Own physical activity behaviour - (anonymous) comparison to group norms	In this lesson, students become aware of their own physical activity behaviour. Based on the anonymous physical activity group mean, students can compare and evaluate their own physical activity behaviour
2	O	- Perceived level of own physical activity - Prepare lesson 3.	Students are asked to give a grade to their own physical activity behaviour (1–10). After this, they are asked why they did not score 2 points lower. The idea here is that students come up with things they *are* doing.
3	C	- Advantages and disadvantages of physical activity and inactivity	The students discuss all advantages and disadvantages of physical activity and inactivity to create ambivalence.
4	O	- Physical activity and sedentary norms - Prepare lesson 4.	Students are made aware of the current physical activity norms (at 60 min of physical activity per day) and sedentary guidelines (less than 2 h of sedentary behaviour per day).
5	C	- Awareness of different qualities of different athletes.	Different athletes are compared by means of Youtube videos. During this lesson, students are made aware that different physical activities require different qualities (e.g. a 100 kg judoka is not a good 100 m distance runner and vice versa).
6	O	- What physical activity suits me?	See also appendix b. this is a table/exercise adapted from the book ‘Bewegen, Sport en Maatschappij’ (physical activity, sports and society) (2008) by Boon, Pecht, Rijper & Stegeman.
7	C	- Action planning	First the students are asked how confident they are to start or commence a physical activity. In the action plan the student describes the what, when, where, and how (what can they do themselves, who do they need, where can they find help) of their physical activity plan.
8	O	- Synthesis of lesson 1–7	Students write a short essay about what they want to do, what they want to achieve, and why.
9	C	- Commitment to the action plan	Students discuss how they will try to achieve their goals, and help each other when necessary.
10	O	- Improvement of action plan	
11	C	Catch up month	
12	C	- Own physical activity behaviour - (anonymous) comparison to group norms - Action	Repetition of lesson 1. In this lesson, the students also have to come up with an idea of what physical activity behaviour they want to start in the coming two months.
13	C	Catch up month/Action month	Students are reminded of lesson 12 and their action plan
14	C	- Experiences and actions	Students discuss (perceived) barriers and solutions to overcome these barriers.
15	C	- Implementation intentions	If-then statements are made to help students to overcome (perceived) barriers.

### Study outcomes

Sex and date of birth were provided by the school’s student administration. Anthropometrics were measured using standard procedures [17]. Height (SECA 213 stadiometer, Hamburg, Germany) and weight (SECA 877 scale, Hamburg, Germany) were measured without shoes or heavy clothes (i.e. only pants and t-shirt) to the nearest 1 mm and 0.1 kg respectively. Body Mass Index (BMI) was calculated as weight/height squared (kg/m^2^) and Z-scores from age- and sex specific reference values [18].

Body composition was assessed by deuterium dilution [19]. After a baseline urine sample, participants drank 75 mL deuterium enriched water, increasing the deuterium body concentration with 70–100 ppm. At least four hours after drinking the deuterium enriched water, the students were required to visit the toilet at least once. This urine was not collected. At the end of the school day (a minimum of 4.5 h later), a second urine sample was collected. To calculate total body water, the two urine samples (baseline and enriched) were analyzed using isotope ratio mass spectrometry [20]. From total body water, fat-free mass was calculated using age-specific hydration fractions [21]. Compared to underwater weighing, deuterium dilution is a valid method to assess fat mass percentage in normal weight and obese subjects [22], showing the same changes in fat free mass over time [23]. Although the technique can be used in all age groups (i.e. accurate, precise, minimal subject cooperation), the method is relatively expensive as it requires on site expertise and expensive analysis equipment. Consequently, it is not often applied in larger studies.

Sedentary behaviour and Physical activity behaviour in daily life was measured using accelerometry (Actigraph GT3x, Actigraph, Pensacola, FL, USA). Students were asked to wear the device on their lower back for five consecutive days, except during sleep and water activities (e.g. taking a shower or swimming). The device was worn on their lower back by using an elastic band [24–26]. The Actilife software (v6.13.3; https://www.actigraphcorp.com/actilife/) was used to generate activity counts (counts per minute; CPM); a higher CPM indicates more physical activity in daily life. Only students who had worn the accelerometer at least 8 h per day during the waking hours (i.e.time awake and time to bed) for a minimum of 3 days were included in the analyses (see Table 4). Wear and non-wear times were determined by the –in Actilife integrated - algorithm of Choi and colleagues [27] and physical activity level cut-off points were determined as proposed by Mattocks and colleagues [28]. The comprehensive intervention study protocol is described elsewhere [15].

### Statistical analysis

Statistical analyses were conducted with IBM SPSS Statistics 20. Outcome variables were body composition (primary outcome fat mass percentage, and secondary outcomes absolute fat mass in kg, and absolute fat free mass in kg), weight, and physical activity (primary outcome was counts per minute, secondary outcomes were percentage of time spent in sedentary behaviour, light physical activity, and moderate to vigorous physical activity using the Mattocks [28] algorithm as integrated in the Actilife software). Mixed (multilevel) regression was used to identify baseline differences between the two conditions on each of the outcomes (with a random school effect on top of the individual pupil effect to adjust for the clustered nature of the data). To test the effectiveness of the intervention mixed regression was used for each outcome variable, using the pretest and posttest as repeated outcome measures, and using time (0 = pretest, 1 = posttest)), condition (0 = control, 1 = intervention), sex (0 = female, 1 = male), age, BMI at baseline, and level of education (0 = Lower Vocational Education, 1 = Senior General Secondary Education) as predictors, plus the interactions of condition, sex, age, BMI and education with time. The covariates sex, age, BMI and level of education and their interaction with time were included for two reasons: First, to increase power by reducing unexplained outcome variance, and secondly, to adjust for possible bias arising from drop out or outcome missingness if related to any of these covariates [29–31]. The random (variance) model part consisted of an unstructured covariance matrix for the within-school variances and covariance of the two repeated measures plus a random intercept for the between-school outcome variance. All participants with at least one measurement (pre or post) were included into the analysis without imputing missing values. This so-called “direct likelihood” method is valid under the same assumptions about missingness as multiple imputation [29]. The initial mixed model additionally contained three-way interactions of time*condition*BMI baseline (and condition*BMI baseline), and time*condition*sex (and condition*sex).^1^ Using Maximum Likelihood estimation and likelihood ratio testing [29] non-significant three-way interactions were removed from the model. Subsequently, non-significant two-way interactions were removed, apart from the condition by time interaction of interest, and any two-way term that was part of a significant three-way term (e.g. age*time if the model still contained condition*age*time). Finally, non-significant (*p* > .05) main effects were deleted, except those that were part of an interaction term in the model (e.g. age if age*time was in the model). All tests were carried out using alpha = 0.05 two-tailed. The final model was rerun with restricted maximum likelihood estimation (REML) to obtain the best estimates of the standard errors of all effects [29]. The model was also rerun after deleting the condition term from the model, which reflects the baseline outcome difference between conditions and should not be significant due to the randomisation. The condition*time effect of interest in this model was compared to that obtained with the final model including the condition term as a robustness check (see van Breukelen [30, 31]).

For all outcome variables, intraclass correlations (ICC) were calculated (per time point) to assess the amount of outcome variance between schools, and normality checks were performed based on the final mixed model (see Additional file 1).

Non-identifiable data, syntax and output of the analyses are fully disclosed (see Additional file 1).

## Results

### Study population

Participant characteristics at baseline are shown in Table 3. No baseline differences were found between the two conditions in age, height, weight, BMI(z), body composition or physical activity outcomes (including wear time of the accelerometer).

**Table 3:** Participant characteristics at baseline

	Total *M* *(SD)*	Control *M* *(SD)*	Intervention *M (SD)*
N	695	342	353
Female:Male	345:350	160:182	185:168
Age (years)	12.97 (0.54)	13.02 (0.54)	12.92 (0.53)
Height (cm)	159.6 (8.2)	160.0 (8.36)	159.3 (8.0)
Weight (kg)	50.4 (11.4)	50.7 (12.0)	50.1 (10.8)
BMI (weight/height in m^2^)	19.7 (3.5)	19.7 (3.8)	19.6 (3.3)
BMI z-score	0.32 (1.18)	0.29 (1.24)	0.36 (1.12)
Education level (N)			
*Lower Vocational Ed.*	561	258	303
*Senior General Secondery Ed.*	134	84	50
Weight status (N[%])^a^			
Underweight	16 (2.3)	9 (2.6)	7 (2.0)
Normal weight	477 (68.6)	231 (67.5)	246 (69.7)
Overweight	140 (20.1)	65 (19.0)	75 (21.2)
Obesity	61 (8.8)	36 (10.5)	25 (7.1)
N	423	199	224
Fat mass (%)	25.1 (8.0)	23.3 (8.2)	26.8 (7.5)
Fat mass (Kg)	13.1 (6.5)	12.0 (6.5)	14.0 (6.3)
Fat Free mass (Kg)	37.3 (7.0)	37.8 (7.4)	36.8 (6.6)
N	460	226	234
Physical Activity (CPM)	677 (200)	675 (189)	679 (209)
Sedentary PA (%)	74.9 (5.6)	75.1 (5.2)	74.8 (6.0)
Light PA (%)	22.8 (4.9)	22.6 (4.5)	23.0 (5.2)
Moderate to Vigorous PA (%)	2.2 (1.4)	2.3 (1.4)	2.2 (1.4)
Accelerometer wear-time T0 (min)	3278 (984)	3340 (926)	3224 (1032)

Note: the analyses of baseline fat and PA measures exclude students whose baseline is missing, but these students are included into the effect analyses if they provide posttest data

^a^ the WHO growth references were used for cut off interpretations: http://www.who.int/growthref/who2007_bmi_for_age/en/

### Missingness analyses

To measure body composition, students handed in two urine samples at school. For the physical activity in daily life measurement, students wore an accelerometer for 5 consecutive days including two weekend days. Due to the nature of these measurements, many students decided not to participate in this measurement (either at T0, T1, or both, see Table 4), resulting in missingness. This is schematically displayed in Table 4; similar patterns of missingness are found in both the control and the intervention group.

**Table 4:** Missingness of body composition measurements

		T1		Total n
		Missing	Not Missing	
Body composition measurement				
Control T0	Missing	119 (35%)	24 (7%)	143 (42%)
	Not Missing	84 (25%)	115 (34%)	199 (58%)
Total n		203 (59%)	139 (41%)	342 (100%)
Intervention T0	Missing	105 (30%)	24 (7%)	129 (37%)
	Not Missing	94 (27%)	130 (37%)	224 (63%)
Total n		199 (56%)	154 (44%)	353 (100%)
Physical activity measurement				
Control T0	Missing	99 (29%)	17 (5%)	116 (34%)
	Not Missing	124 (36%)	102 (30%)	226 (66%)
Total n		223 (65%)	119 (35%)	342 (100%)
Intervention T0	Missing	104 (29)	15 (4%)	119 (34%)
	Not Missing	130 (37%)	104 (29%)	234 (66%)
Total n		234 (66%)	119 (34%)	353 (100%)

See section 3.2 for an explanation how missingness was handled in the analyses

Participants with missing data at T0 *and* T1 could not be included in the mixed regression effect analysis. To check possible bias arising from this, the relation of such complete missingness (i.e. missing at T0 and T1 simultaneously) to condition, age, sex, level of education, baseline BMI, and the ‘switch’ variable^1^ was checked with logistic regression (results are summarised in Additional file 1). Possible bias arising from how these baseline variables relate to complete missingness of an outcome was resolved by including all baseline variables as predictors into the effect analyses with mixed regression [29]. The following baseline variables were related to complete missingness: ‘switch’ (*p* = .04 for body composition measurement and *p* < .01 for the physical activity measurement), level of education (*p <* .01 for physical activity measurement), and sex (*p <* .001 for physical activity measurement).

### Effect of the intervention on body composition

**Fig. 2:**
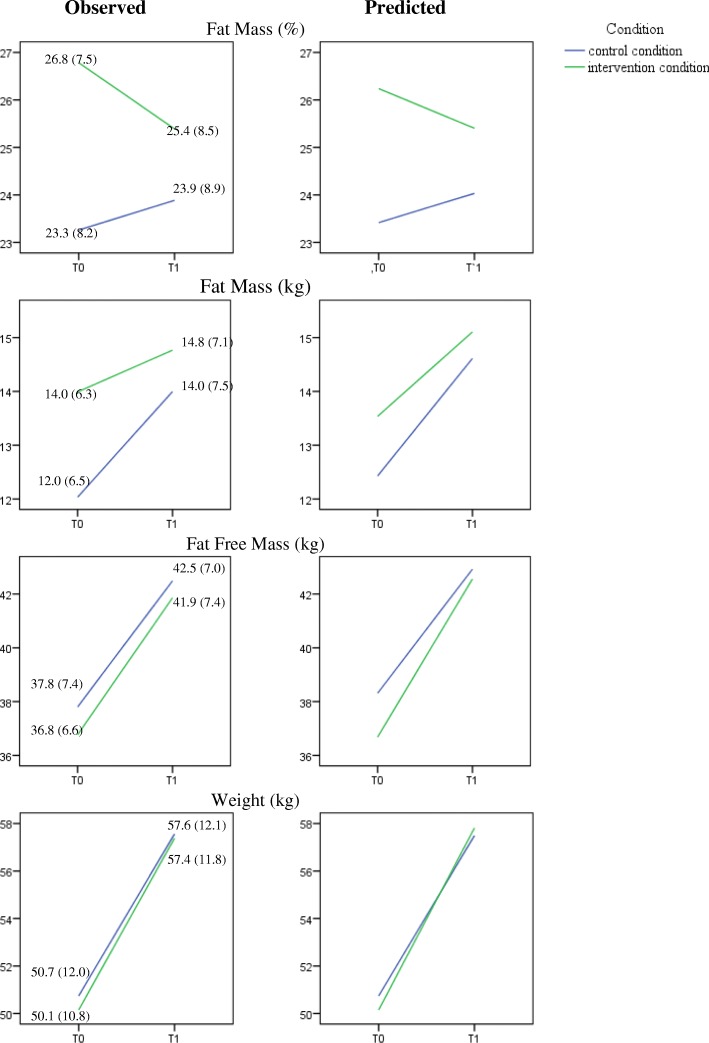
Effect of the intervention on body composition. Body composition scores as measured by the deuterium dilution technique: Observed data, possibly biased by missingness (left panel), and predicted means based on the mixed regression (right panel). Note that observed means and SDs can be biased by ignoring both the clustering and dropout/missingness. The predicted means based on mixed regression are the best estimates of the time courses of all outcomes

After one year, a 1.6% fat mass difference was found in favour of the intervention group (*p* = .007); see Fig. 2 and Table 5. In absolute terms, this reflected a 0.9 kg difference in fat free mass (intervention condition >control condition; *p* = .041) and 0.7 kg in fat mass (intervention condition < control condition; *p* = .054). Furthermore, and apart from these intervention effects, boys had a significantly lower fat mass percentage and absolute fat mass, and higher absolute fat free mass than girls at pretest (see the Sex-effect in Table 3). At posttest, these differences had further increased for fat mass percentage (− 3.7%, *p* < .001), absolute fat mass (− 1.9 kg, *p <* .001), and absolute fat free mass (+ 3.9 kg, *p* < .001), see the time by sex effect in Table 3. However, these differences were equal in both conditions (no time*sex*condition effect).

**Table 5:** Outcomes of the mixed multilevel regression models for dependent variable fat mass percentage, absolute fat mass (kg), absolute fat free mass (kg), and body weight^a^

	Fat Mass %		Fat Mass (Kg)		Fat Free Mass (Kg)		Body Weight (Kg)^c^			
							Boys^c^		Girls^c^	
	Estimate	95% CI	Estimate	95% CI	Estimate	95% CI	Estimate	95% CI	Estimate	95% CI
	*(SE)*		*(SE)*		*(SE)*		*(SE)*		*(SE)*	
Intercept	−17.81 (6.56)**	4.9–30.7	− 17.79 (1.04)***	− 19.9 – − 15.7	−20.99 (6.17)**	−33.1 – − 8.9	−42.51 (7.03)***	−56.3 – − 28.7	−20.23 (5.82)***	−31.7 – − 8.8
Time^b^	1.35 (1.89)	−2.4– 5.1	− 0.99 (1.10)	− 3.2 – 1.2	26.00 (6.13)***	13.9–38.1	23.60 (8.59)**	6.7–40.5	32.37 (6.76)***	19.1–45.7
Condition^b^	2.83 (1.02)*	0.1–5.6	1.45 (0.78)	−0.5 – 3.4	−1.25 (0.77)	−3.4 – 0.9	− 0.36 (0.58)	−1.5 – 0.8	−0.00 (0.72)	− 1.8 – 1.8
Age	−1.57 (0.50)**	−2.6 – − 0.6	–	–	2.62 (0.47)***	1.7–3.5	2.67 (0.54)***	1.6–3.7	1.28 (0.45)**	0.4–2.2
Sex^b^	− 2.94 (0.56)***	−4.0 – − 1.8	−1.37 (0.30)***	−2.0 – − 0.8	1.81 (0.50)***	0.8–2.8	–	–	–	–
Level of education	–	–	–	–	–	–	–	–	–	–
BMI baseline	1.39*(0.08)**	1.2–1.6	1.55 (0.04)***	1.5–1.6	1.23 (0.07)***	1.1–1.4	2.98 (0.83)***	2.8–3.1	2.74 (0.07)***	2.6–2.9
Time*Condition	−1.59 (0.58)**	−2.7 – − 0.4	−0.69 (0.36)	−1.4 – 0.0	0.93 (0.45)*	0.0–1.8	1.47 (0.63)*	0.2–2.7	−0.64 (0.53)	−1.7 – 0.4
Time*Age	–	–	-	–	−1.66 (0.44)***	−2.5 – −0.8	− 1.35 (0.59)*	−2.5 – − 0.2	−1.92 (0.48)***	−2.9 – − 1.0
Time *Sex	−3.66 (0.58)***	−4.8 – − 2.5	−1.86 (0.36)***	−2.6 – − 1.1	3.90 (0.46)***	3.0–4.8	–	–	–	–
Time*BMI baseline	0.17 (0.09)	−0.0 – 0.3	0.21 (0.05)***	0.1–0.3	−0.14 (0.07)*	− 0.3 – − 0.1	–	–	–	–

Intraclass correlations (ratio of between-school variance to between+within-school variance) were (all between 0.01–0.10, for FM% (primary outcome): ICC = .04 at both T0 and T1)

*p < .05; **p < .01; ***p < .001

^a^ The variables ‘Switch’ and switch x time were in the initial model (see Sample size and statistical analyses section, Footnote a). As these were not significant, these were excluded in the clean model as described in the table

^b^ Coding: Time (0 = T0, 1 = T1), Condition (0 = Control condition, 1 = Intervention condition), Sex (0 = girls, 1 = boys)

^c^ For weight, a time*condition*sex effect was found (*p* = .005). Therefore, the effects for boys and girls are displayed separately in this table. The three-way interaction can be found in Additional file 1

A three-way interaction was found for the effects of the intervention on weight (Time*condition*sex, see Additional file 1). The same analysis but split on sex showed that body weight increased substantially from pre- to posttest for boys and girls in both conditions. However, for boys the increase was 1.5 kg more in the intervention condition than in the control condition (*p =* .019), partly due to an increase in fat free mass (*p* = .07). For girls, no significant difference (with respect to change) was found between conditions (− 0.6 kg, *p* = .22). Rerunning all final models without the condition term (which represents the baseline difference between conditions and must be zero apart from chance differences due to the randomization) gave very similar condition*time effects as the final models themselves, indicating robustness of these effects against baseline differences [30, 31](see Additional file 1).

### Effect of the intervention on physical activity behaviour in daily life

**Fig. 3:**
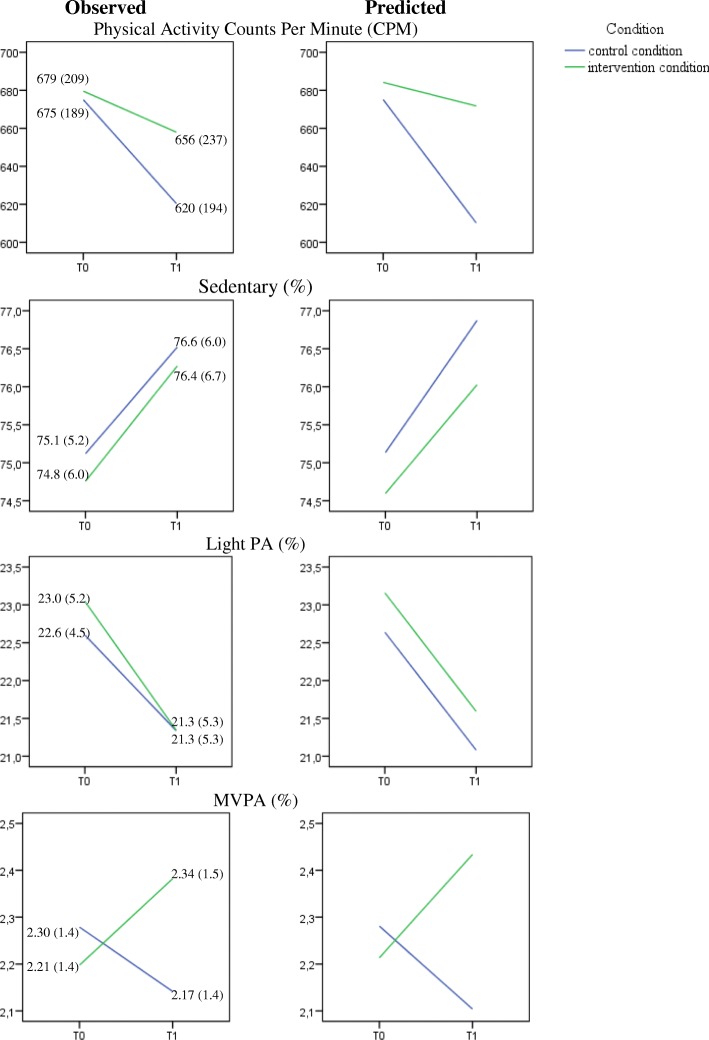
Effect of the intervention on physical activity. Physical activity measured by accelerometers: Observed data, possibly biased by missingness (left panel), and predicted means based on the mixed regression (right panel). Note that observed means and SDs can be biased by ignoring both the clustering and dropout/missingness. The predicted means based on mixed regression are the best estimates of the time courses of all outcomes

Daily PA decreased from baseline to posttest in both groups, but less so in the intervention group (*p* = .049; see Fig. 3 and Table 6). The time-effect was not significant in the intervention-group (*p* = .490). After 1 year, no differences in sedentary behaviour (*p* = .715), or light physical activity (*p* = .833) were found between groups. After 1 year, a small but significant difference of 0.4% was found for moderate to vigorous physical activities in favour of the intervention group (*p* = .046; this can be translated to approximately 6 min per day). Rerunning the final models without the condition term did not substantially change the results for the condition*time effect of interest [30, 31] (see Additional file 1).

**Table 6:** Outcomes of the mixed multilevel regression models for dependent variable physical activity (in counts per minute; CPM), sedentary behavior (%), light physical activity (%), and moderate to vigorous physical activity (%)^a^

	Physical Activity (CPM)		Physical Activity		Physical Activity		Physical Activity	
			Sedentary %		Light %		Moderate to Vigorous %	
	Estimate	95% CI	Estimate	95% CI	Estimate	95% CI	Estimate	95% CI
	*M (SE)*	*M (SE)*	*M (SE)*	*M (SE)*	*M (SE)*	*M (SE)*	*M (SE)*	*M (SE)*
Intercept	807.34 (58.28)***	692–923	72.32 (1.70)	68.9–75.7	23.25 (0.68)***	21.7–24.8	3.25 (0.38)***	2.5–4.0
Time^b^	− 63.22 (18.31)***	−99 – − 27	1.71 (0.56)	0.6–2.8	−1.52 (0.50)**	−2.5 – − 0.5	− 0.16 (0.13)	−0.4 –0.1
Condition^b^	− 8.91(40.95)	−107 – 88	0.16 (1.26)	−2.8 – 3.2	0.03 (0.91)	−2.2 – 2.2	−0.14 (0.24)	− 0.7 –0.4
Age	–	–	–	–	–	–	–	–
Sex^b^	66.46 (16.99)***	33–100	−0.97 (0.49)	− 1.8 – − 0.0	–	–	0.45 (0.11)***	0.2–0.7
Level of education	–	–	–	–	−2.20 (1.07)	− 4.8 – 0.4	–	–
BMI baseline	−7.98 (2.48)***	−13 – 3	0.15 (0.07)	0.0–0.3	–	–	− 0.06 (0.02)***	−0.1 – − 0.0
Time*Condition	50.99 (25.75)*	0–100	−0.29 (0.78)	−1.8 – 1.3	− 0.15 (0.70)	− 1.5 – 1.2	0.38 (0.19)*	0.0–0.8

Intraclass correlations (all between 0.01–0.10

*p < .05; **p < .01; ***p < .001

^a^ The variables ‘Switch’, ‘Time*Switch’ (Footnote a), “Time*Sex’, ‘Time*BMI baseline’ and ‘Time*Level of education” were in the initial model. As these were not significant, these are excluded in the clean model as described in the table

^b^ Coding: Time (0 = T0, 1 = T1), Condition (0 = Control condition, 1 = Intervention condition), Sex (0 = girls, 1 = boys

## Discussion

### Study outcomes

In a school-based randomised controlled trial, we found that small and easily implementable changes in secondary schools (minor adjustments to the physical education classes, plus physical activity motivation) resulted in favourable differences in body composition in 11–15 year-old adolescents. After one year, a 1.6% fat mass difference was found in favor of the intervention group, reflecting a 0.9 kg difference in fat free mass (intervention condition >control condition) and 0.7 kg in fat mass (intervention condition <control condition). With respect to physical activity, all adolescents became less physically active after 1 year. This is a phenomenon that is seen more often in youth [32, 33]. However, due to the intervention, a lower decrease in physical activity level was found in the intervention group compared to the control group. The improved body composition, and less decrease in physical activity might positively influence short term and long term metabolic health and chronic health risks.

The beneficial effects of improving body composition are twofold. First, it is well known that a higher fat percentage is related to several cardiovascular risk factors, such as increased triglyceride levels, higher LDL, lower HDL, increased blood pressure and insulin resistance, and that these associations already exist in children and adolescents [15]. Secondly, increasing fat-free mass not only increases basal metabolic rate -- but also total energy expenditure [34].

Our results also indicate that the use of strength training at school alongside a motivational intervention can induce a change in activity levels, also outside physical education classes. Several reviews have shown a dose-response relationship between physical activity and health showing that any improvement in physical activity behaviour may have beneficial effects [35].

### Strength training as a new approach

In this study, we bridged the gap between biological and psychological approaches to the management of obesity, showing that strength exercises have both physiological and psychological benefits for adolescents who are overweight or obese. By focusing on the general first year secondary school population, and not only on overweight or obese youngsters, we attempted to minimise any stigma associated with obesity and encourage interpersonal appreciation.

Our aim was not to reduce obesity per se, but to tackle obesity-related health issues. Our approach focuses on making overweight youngsters healthier (in terms of body composition) and motivating them to be more physically active. This was achieved, not by focusing on BMI, or the idea that overweight youngsters have to lose weight, but rather by focusing on their strength and on what overweight youngsters like to do.

### Reflection on earlier studies

An important key term in our study was social comparison: the optimal use of the comparison of overweight youngsters with normal-weight youngsters. Where the strength of overweight and obese youngsters compared to normal weight youngsters is their (absolute) muscle strength, very often aerobic exercises or relative muscle strength exercises are offered (as pragmatic, resource or class management decision), leading to a decreased motivation (see also Table 1). There are limited studies available when it comes to strength exercises in a school based setting. In a study by Smith et al. [36], a school-based program was conducted. In their 20-week intervention, 361 adolescents who were considered at risk of obesity followed a similar intervention, but no results were found on BMI, waist circumference, body fat or physical activity. An important difference with their study was the body composition measurement (bioelectrical impedance analyses) they used. Although the authors suggest that the inclusion of healthy weight participants might have minimized their effects, we argue that the inclusion of normal weight participants might be beneficial in terms of social comparison. In a recent study by Kennedy et al. [37], the effects of strength training on muscular fitness was examined. This muscular fitness were examined by push up tests and a standing long jump test. They found improvements for the upper body, but not the lower body. After six months, effects of strength training on skill competency and self efficacy were found, but not on BMI, flexibility, physical activity, or motivation. Although the validity and reliability of the tests used in their study is high, in our study we argued that adolescents who are overweight or obese have a higher absolute muscle strength, and perform better in absolute strength exercises. Relative exercises (and tests) are less beneficial for adolescents who are overweight or obese in terms of social comparison (they perform less compared to normal weight youngsters on these exercises) and with that, in terms of motivation to exercise..

### Process evaluation, diffusion and implementation

We have shown that strength exercises during physical education classes in secondary schools improve body composition and probably promote physical activity. At this point, it is difficult to estimate the magnitude of long-term effects. Replications of these findings are needed, but also diffusion and implementation. We collected process evaluation data on secondary physiological and psychological outcomes, and are collecting data on teacher experiences with our programme (not reported here); both will result in further suggestions to improve the intervention. During the intervention period, the online motivational lessons were monitored to see the students progress (all schools finished the online lessons). For the motivational lessons in classical setting and the strength exercise lessons, the teachers were asked to evaluate their progress halfway, and at the end of the intervention. Teachers reported an average-to-good program delivery. The basic idea is simple and easily implementable. Infrastructures at schools can be optimised. Physical education teachers can be informed about strength exercises, and provided with guidelines and suggestions for practice [38]. A work book with exercises is freely available (see Additional file 1), but it was noticed that teachers themselves can easily come up with new ideas about strength exercises in the lessons the moment they understand the principle and find out that the students react positively, especially the students with overweight.

Outside the school setting, different sports in which pure physical strength and/or body mass are beneficial (eg. rugby, judo) could be systematically promoted as an alternative for youngsters who are overweight. Fitness centres offer strength training, but they are often inaccessible for youngsters, suggesting that a collaboration between schools and fitness centres may be fruitful. Schools could consider providing this equipment. Additionally, we recommend increasing parental awareness of the advantages of strength training in terms of their child’s health [10, 11].

### Strengths and limitations

In this study we used highly accurate measuring techniques (deuterium dilution technique and accelerometry) in a relatively difficult setting (high schools). Although both measuring techniques (deuterium dilution and accelerometry) are acceptable in all age groups, the method is relatively expensive and thus often not applied in larger studies. Due to the nature of these measurements, many students decided to not participate in this measurement (either at T0, T1, or both, see Table 4 for exact numbers), resulting in missingness. While all available data were included into the analysis using a method that is valid under so-called missingness at random (MAR, missingness depends on observed variables such as age or pretest if posttest is missing), we cannot rule out bias arising from missingness not at random (MNAR, missingness depends on unobserved variables such as posttest if posttest is missing). Unfortunately, MNAR cannot be detected or adjusted for. At best, complex methods can be used to explore the robustness of results against various patterns of MNAR missingness [29]. Further, although our sample size was quite large, our study was underpowered both due to the larger than expected dropout or missingness and due to the clustered data structure (students nested within schools, randomization of schools). It is encouraging that, in spite of that low power, both primary outcomes showed a significant and beneficial intervention effect. It is unclear to what extent the absence of a significant effect on various other outcomes is due to low power. Lastly, we did not measure sitting height, so we were unable to calculate maturation. This might be of additional value in future studies.

## Conclusion

In this study, we implemented and evaluated an interdisciplinary theory- and evidence-based program that positively influenced body composition and physical activity. This might not be a direct solution to combat obesity, but it might help in the long term with the prevention of obesity related health issues. We do not want children and adolescents to become extremely muscular, nor should aerobic components be banned. Rather, we recommend that strength exercises, under qualified supervision should be added to a child’s physical activity routine: as long as strength exercises are performed under qualified supervision, they might have positive long term health benefits [39].

## Additional file

Additional file 1: Full disclosure of materials, data, analyses, and output. (RAR 16265 kb)

## Abbreviations

BMI: Body Mass Index
CPM: Counts per Minute
ICC: Intraclass Correlation
MAR: Missingness at Random
MNAR: Missingness Non at Random
PE: Physical education
REML: Restricted maximum likelihood estimation

## Footnotes

1 Following baseline measurements, we found out that in one of the schools, 3 out of 5 classes that had been allocated to the intervention condition were going to be transferred (after five months) to another school that had been allocated to the control condition. To minimise the chance of contamination, these 3 classes did not receive the intervention. To check the effect of this protocol deviation on the outcome variables, an extra predictor was added to the model which indicated this treatment switch (0 = not switched, 1 = switched), and its interaction with time was added as well. Neither that interaction nor the main effect of switch after dropping the interaction from the model was significant for any outcome, probably due to low power resulting from the fact that outcome measurements were missing for most switchers anyway. The reported effect analyses therefore do not adjust for switch, thus complying with the intention to treat principle (for further details of missingness, see the Results section).

## References

[1] NCD Risk Factor Collaboration (2017). Worldwide trends in body-mass index, underweight, overweight, and obesity from 1975 to 2016: a pooled analysis of 2416 population-based measurement studies in 128 9 million children, adolescents, and adults. Lancet.

[2] Swinburn BA, Sacks G, Hall KD, McPherson K, Finegood DT, Moodie ML (2011). The global obesity pandemic: shaped by global drivers and local environments. Lancet.

[3] Kelsey MM, Zaepfel A, Bjornstad P, Nadeau K (2014). Age-related consequences of childhood obesity. Gerontology.

[4] Washington RL (2008). Metabolic syndrome – no longer an adult only disease. J Pediatr.

[5] Hunger JM, Tomiyama AJ (2014). Research letter: weight labeling and obesity: a longitudinal study of girls aged 10 to 19 years. JAMA Pediatr.

[6] https://www.volksgezondheidenzorg.info/onderwerp/overgewicht/cijfers-context/huidige-situatie#node-overgewicht-kinderen, Accessed: 20/11/2017.

[7] Kremers SP, Visscher TL, Seidell JC, van Mechelen W, Brug J (2005). Cognitive determinants of energy balance-related behaviours: measurement issues. Sports Med.

[8] Lee IM, Shiroma EJ, Lobelo F, Puska P, Blair SN, Katzmarzyk PT (2012). Effect of physical inactivity on major non-communicable diseases worldwide: an analysis of burden of disease and life expectancy. Lancet.

[9] Ochner CN, Barrios DM, Lee CD, Pi-Sunyer FX (2013). Biological mechanisms that promote weight regain following weight loss in obese humans. Physiol Behav.

[10] Ten Hoor GA, Plasqui G, Schols AM, Kok G (2014). Combating adolescent obesity: an integrated physiological and psychological perspective. Curr Opin Clinl Nutr.

[11] Ten Hoor GA, Plasqui G, Ruiter RAC, Kremers SPJ, Rutten GM, Schols AMWJ (2016). A new direction in psychology and health: resistance exercise training for obese children and adolescents. Psychol Health.

[12] Freedman DS, Ogden CL, Kit BK (2015). Interrelationships between BMI, skinfold thicknesses, percent body fat, and cardiovascular disease risk factors among US children and adolescents. BMC Pediatr.

[13] Moran Jason, Sandercock Gavin, Ramirez-Campillo Rodrigo, Clark Cain C. T., Fernandes John F. T., Drury Benjamin (2018). A Meta-Analysis of Resistance Training in Female Youth: Its Effect on Muscular Strength, and Shortcomings in the Literature. Sports Medicine.

[14] van Breukelen GJ, Candel MJ (2012). Calculating sample sizes for cluster randomized trials: we can keep it simple and efficient!. J Clin Epidemiol.

[15] Ten Hoor GA, Kok G, Rutten GM, Ruiter RAC, Kremers SPJ, Schols AMWJ, et al. The Dutch 'Focus on Strength'intervention study protocol: programme design and production,implementation and evaluation plan. BMC Public Health. 2016;16(1). 10.1186/s12889-016-3150-6. 10.1186/s12889-016-3150-6 PMC4902907 27287848

[16] Rollnick S, Kaplan SG, Rutschman R (2016). Motivational Interviewing in Schools: Conversations to Improve Behavior and Learning.

[17] National Health and Nutrition Examination Survey, III, 1988–94. National Center for Health Statistics. Rockville: Center for Disease Control and Prevention (CDC); 1997. https://wwwn.cdc.gov/nchs/nhanes/nhanes3/default.aspx.

[18] Fredriks AM, Van Buuren S, Wit JM, Verloove-Vanhorick SP (2000). Body index measurements in 1996–7 compared with 1980. Arch Dis Child.

[19] Westerterp KR, Wouters L, van Marken LW (1995). The Maastricht protocol for the measurement of body composition and energy expenditure with labeled water. Obes Res.

[20] Schoeller DA, Ravussin E, Schutz Y, Acheson KJ, Baertschi P, Jequier E (1986). Energy expenditure by doubly labeled water: validation in humans and proposed calculation. Am J Physiol-Reg I.

[21] Lohman TG (1989). Assessment of body composition in children. Pediatr Exerc Sci.

[22] WESTERTERP KLAAS R., MEIJER GERWIN A. L., SARIS WIM H. M., SOETERS PETER B., WINANTS YVONNE, HOOR FOPPE TEN (1991). Physical activity and sleeping metabolic rate. Medicine & Science in Sports & Exercise.

[23] Van der Kooy K, Leenen R, Deurenberg P, Seidell JC, Westerterp KR, Hautvast JG (1992). Changes in fat-free mass in obese subjects after weight loss: a comparison of body composition measures. Int J Obes Relat Metab Disord.

[24] Plasqui G, Bonomi A, Westerterp KR (2013). Daily physical activity assessment with accelerometers: new insights and validation studies. Obes Rev.

[25] Plasqui G (2017). Smart approaches for assessing free-living energy expenditure following identification of types of physical activity. Obes Rev.

[26] Yngve A, Nilsson A, Sjostrom M, Ekelund U (2003). Effect of monitor placement and of activity setting on the MTI accelerometer output. Med Sci Sports Exerc.

[27] CHOI LEENA, LIU ZHOUWEN, MATTHEWS CHARLES E., BUCHOWSKI MACIEJ S. (2011). Validation of Accelerometer Wear and Nonwear Time Classification Algorithm. Medicine & Science in Sports & Exercise.

[28] Mattocks Calum, Leary Sam, Ness Andy, Deere Kevin, Saunders Joanne, Tilling Kate, Kirkby Joanne, Blair Steven N., Riddoch Chris (2007). Calibration of an accelerometer during free-living activities in children. International Journal of Pediatric Obesity.

[29] Verbeke G, Molenberghs G (2000). Linear mixed models for longitudinal data.

[30] Van Breukelen GJP (2013). ANCOVA versus change from baseline in nonrandomized studies: the difference. Multivar Behav Res.

[31] Van Breukelen GJP (2006). ANCOVA versus change from baseline: more power in randomized studies, more bias in nonrandomized studies. J Clin Epidemiol.

[32] Ruiz JR, Ortega FB, Martinez-Gomez D, Labayen I, Moreno LA, De Bourdeaudhuij I (2011). Objectively measured physical activity and sedentary time in European adolescents: the HELENA study. Am J Epidemiol.

[33] Van Mechelen W, Twisk JW, Post GB, Snel J, Kemper HC (2000). Physical activity of young people: the Amsterdam longitudinal growth and health study. Med Sci Sports Exerc.

[34] Plasqui G, Joosen AM, Kester AD, Goris AH, Westerterp KR (2005). Measuring free-living energy expenditure and physical activity with Triaxial Accelerometry. Obes Res.

[35] Janssen Ian, LeBlanc Allana G (2010). Systematic review of the health benefits of physical activity and fitness in school-aged children and youth. International Journal of Behavioral Nutrition and Physical Activity.

[36] Smith JJ, Morgan PJ, Plotnikoff RC, Dally KA, Salmon J (2014). Smart-phone obesity prevention trial for adolescent boys in low-income communities: the ATLAS RCT. Pediatrics.

[37] KENNEDY SARAH G., SMITH JORDAN J., MORGAN PHILIP J., PERALTA LOUISA R., HILLAND TONI A., EATHER NARELLE, LONSDALE CHRIS, OKELY ANTHONY D., PLOTNIKOFF RONALD C., SALMON JO, DEWAR DEBORAH L., ESTABROOKS PAUL A., POLLOCK EMMA, FINN TARA L., LUBANS DAVID R. (2018). Implementing Resistance Training in Secondary Schools. Medicine & Science in Sports & Exercise.

[38] Lloyd RS, Faigenbaum AD, Stone MH, Oliver JL, Jeffreys I, Moody JA (2014). Position statement on youth resistance training: the 2014 international consensus. Br J Sports Med.

[39] Faigenbaum AD, Kraemer WJ, Blimkie CJ, Jeffreys I, Micheli LJ (2009). Youth resistance training: updated position statement paper from the national strength and conditioning association. J Strength Cond Res.

